# Go Play Outside! Effects of a risk-reframing tool on mothers’ tolerance for, and parenting practices associated with, children’s risky play: study protocol for a randomized controlled trial

**DOI:** 10.1186/s13063-018-2552-4

**Published:** 2018-03-07

**Authors:** Mariana Brussoni, Takuro Ishikawa, Christina Han, Ian Pike, Anita Bundy, Guy Faulkner, Louise C. Mâsse

**Affiliations:** 1 Department of Pediatrics, School of Population and Public Health, University of British Columbia, British Columbia Injury Research and Prevention Unit, British Columbia Children’s Hospital Research Institute, F511–4480 Oak Street, Vancouver, BC V6H 3V4 Canada; 2 Department of Pediatrics, University of British Columbia, British Columbia Injury Research and Prevention Unit, British Columbia Children’s Hospital Research Institute, F508–4480 Oak Street, Vancouver, BC V6H 3V4 Canada; 3 Occupational Therapy, Colorado State University, Faculty of Health Sciences, University of Sydney, 1573 Campus Delivery, Fort Collins, CO 80523 USA; 4 School of Kinesiology, University of British Columbia, D.H. Copp Building, Room 4606, 2146 Health Sciences Mall, Vancouver, BC V6T 1Z3 Canada; 5 School of Population and Public Health, University of British Columbia, British Columbia Injury Research and Prevention Unit, British Columbia Children’s Hospital Research Institute, F508–4480 Oak Street, Vancouver, BC V6H 3V4 Canada

**Keywords:** Outdoor play, Mothering, Independent mobility, Physical activity, Risk perception

## Abstract

**Background:**

Children’s risky play is associated with a variety of positive developmental, physical and mental health outcomes, including greater physical activity, self-confidence and risk-management skills. Children’s opportunities for risky play have eroded over time, limited by parents’ fears and beliefs about risk, particularly among mothers. We developed a digital tool and in-person Risk-reframing (RR) workshop to reframe parents’ perceptions of risk and change parenting behaviours. The purpose of this paper is to describe our RR intervention, rationale and protocol for a randomised controlled trial to examine whether it leads to increases in mothers’ tolerance of risk in play and goal attainment relating to promoting their child’s opportunities for risky play.

**Methods:**

We use a randomised controlled trial design and will recruit a total of 501 mothers of children aged 6–12 years. The RR digital tool is designed for a one-time visit and includes three chapters of self-reflection and experiential learning tasks. The RR in-person tool is a 2-h facilitated workshop in which participants are guided through discussion of the same tasks contained within the digital tool. The control condition consists of reading the Position Statement on Active Outdoor Play.

Primary outcome is increased tolerance of risk in play, as measured by the Tolerance of Risk in Play Scale. Secondary outcome is self-reported attainment of a behaviour-change goal that participants set for themselves.

We will test the hypothesis that there will be differences between the experimental and control conditions with respect to tolerance of risk in play using mixed-effects models. We will test the hypothesis that there will be differences between the experimental and control conditions with respect to goal attainment using logistic regression.

**Discussion:**

The results of this trial will have important implications for facilitating the widespread change in parents’ risk perception that is necessary for promoting broad societal understanding of the importance of children’s risky play. In addition, the findings may provide relevant information for the design of behaviour-change tools to increase parental tolerance of risk.

**Trial registration:**

ClinicalTrials.gov, ID: NCT03374683. Retrospectively registered on 15 December 2017.

**Electronic supplementary material:**

The online version of this article (10.1186/s13063-018-2552-4) contains supplementary material, which is available to authorized users.

## Background

An abundance of evidence points to the fundamental importance of play for children’s development and wellbeing [1]. More recently, opportunities for unstructured play have been linked to children’s mental and physical health, including promoting physical activity, mental wellbeing, and executive function [2–4]. In particular, the importance of outdoor play for promoting physical activity has been recently capitalised upon by numerous governments and public health campaigns in order to address the growing obesity epidemic [5–8].

The opportunity to engage with risk is a fundamental part of play. Typically occurring outdoors, risky play involves experimenting with uncertainty and overcoming fears [9]. Sandseter [9] outlines six categories of risky play, including play at speed (e.g. game of chase), heights (e.g. climbing trees), with tools (e.g. building a fort), near dangerous elements (e.g. fire, water, cliffs) and venturing out without adults (e.g. walking to school with friends). Scholarship investigating risky play has intensified in the last decade and has identified that exposure to risky play can help children to gain mastery, promote self-confidence and social skills, and reduce anxiety and depression [10, 11]. A systematic review found positive associations with physical activity and social health, negative associations with sedentary behaviour, and no association with injury risk [12]. Four- and 6-year-old children participating in a 14-week intervention that involved engaging in activities reflecting the different types of risky play improved their reaction time in detecting risk, increased self-esteem and decreased conflict sensitivity relative to their pre-intervention performance, as well as when compared to an age-matched control group [13]. A cross-sectional study compared 5-year-olds with and without ready access to unsupervised outdoor play opportunities and found more developed motor skills, social behaviour, independence and conflict resolution in the former group [14]. Furthermore, experience with risk during childhood is believed to assist with developing risk-management strategies, and the ability to negotiate decisions about substance use, relationships and sexual behaviour during adolescence [15, 16].

Children’s independent mobility, which refers to their freedom to travel around their neighbourhood by themselves without adult supervision, is one example of outdoor risky play and may be important for facilitating other opportunities for risky play [9]. However, opportunities for outdoor risky play regarding independent mobility have been increasingly eroded over time. In England, the percentage of children aged 7–11 years who were allowed to travel to school alone in 1971 was 86%. This dropped to 35% in 1990 and then to 25% in 2010 [17]. Australian research reported that 12% of Australian children aged 8–12 years were not permitted to go anywhere without an adult, and 32% had an independent mobility range of less than one block [18]. Canadian research with 9–13-year-old children found that on average 94.5% of the participants’ time was spent less than 400 m from their homes and that they spent only a very small portion of their time in the larger neighbourhood context [19].

Numerous international studies have identified that parental fears, attitudes about social dangers, and perceptions of the value of free play and outdoor autonomy exert a strong influence on children’s outdoor risky play opportunities [20–28]. In a 16-nation study, traffic safety concerns, followed by fear of strangers, were the strongest factors investigated that influenced parents’ decisions regarding children’s independent mobility [29]. Children’s risk taking in play is also limited by fear of serious injury and of disapproval and censure from other parents and adults [30, 31]. Such parental anxieties have led to a ‘backseat’ and ‘bubble-wrapped’ generation, relying on automobile-based commuting and little unstructured outdoor play time [26, 32].

Societal shifts to promote children’s outdoor risky play may benefit by reframing parents’ beliefs about risk, which in turn may reduce anxiety-based caregiving. In particular, because mothers typically express greater concerns and place more limits on children’s activities than fathers, they are an important target audience for efforts to promote change [33, 34]. Bundy and colleagues developed the Risk-reframing (RR) workshop, a 2-h, in-person group session in which parents and educators are led through a series of reflection points designed to change attitudes and behaviours related to children’s outdoor play [21, 35]. Its effectiveness has been previously documented [36, 37]. However, the workshop format and length have significant resource implications, limiting its availability and amenability to large-scale distribution.

Digital tools for health-behaviour change have become increasingly popular as vehicles for intervention delivery. They can provide an efficacious, convenient and cost-effective means of combining broad reach with the tailored approach of in-person interventions [38, 39]. Using Bundy et al.’s [21, 35] RR workshop as a starting point, we used the principles of health-behaviour change and social cognitive theory to develop a RR intervention to reframe parents’ attitudes and behaviours about their children’s outdoor risky play [40, 41] consisting of an RR digital tool (https://OutsidePlay.ca), and an in-person facilitated workshop (with PowerPoint slides and facilitator manual). Having two versions of the RR intervention may help maximise the reach and flexibility of the intervention, such that it can be accessed independently by anyone and be shared broadly with their network in addition to being offered as a workshop with a standardised delivery protocol by organisations, schools and recreation providers that work with parents. The purpose of this paper is to describe the RR intervention, our rationale and protocol for a randomised controlled trial to evaluate whether it influences mothers’ attitudes and self-reported behaviours relating to facilitating their child’s opportunities for risky play.

### Study aims, research questions, and hypotheses

Our aim is to assess the effectiveness of the RR intervention to increase mothers’ tolerance for risky play and attain a behaviour-change goal relating to providing risky play opportunities for their 6–12-year-old children. We will test the two versions of the intervention:

1. The RR digital tool
2. The RR in-person, 2-h workshop

We hypothesise that:

1. Mothers completing the RR digital tool will have a significantly greater increase of tolerance for risk in play than mothers in the control condition
2. Mothers completing the RR in-person workshop will have a significantly greater increase of tolerance for risk in play than mothers in the control condition
3. A greater proportion of mothers completing the RR digital tool will attain their behaviour-change goal, than mothers in the control condition
4. A greater proportion of mothers completing the RR in-person workshop will attain their behaviour-change goal than mothers in the control condition

## Methods

### Study design

**Fig. 1:**
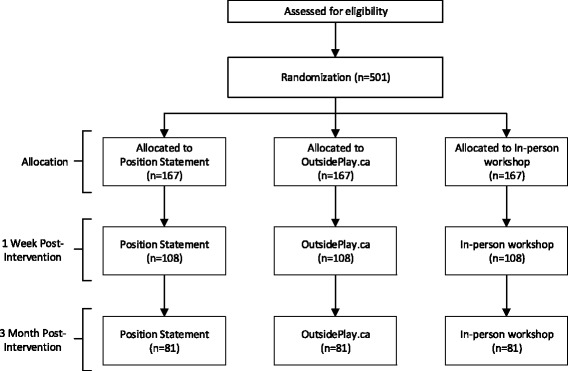
Study flow chart

The study uses a single-blind (researchers and outcome assessors), three-parallel-group randomised controlled trial (RCT) design, to determine the superior effect of the RR digital tool and the RR in-person workshop over the control condition. The trial was retrospectively registered on 15 December 2017 with the United States National Institute of Health’s Protocol Registration and Results System at https://clinicaltrials.gov (NCT03374683). The study flow chart can be seen in Fig. 1 and the Standard Protocol Items: Recommendations for Interventional Trials (SPIRIT) Checklist is available as Additional file 1. Scientific lead, study management and coordination, participant recruitment, data collection and statistical analyses are performed by the British Columbia Children’s Hospital Research Institute, University of British Columbia.

Once participants are deemed eligible for the study, they are automatically allocated to one of the three conditions by the REDCap electronic data capture tool hosted at British Columbia Children’s Hospital Research Institute [42]: (1) control; (2) the RR digital tool; and (3) the RR in-person workshop. Participants in Condition 1 will be provided with a link to the Position Statement on Active Outdoor Play, which includes information on research and recommendations for action [43, 44]. Participants in Condition 2 will be provided with a link to the RR digital tool to complete within the next week. Participants in Condition 3 will be scheduled to attend the RR in-person workshop. The randomisation schedule was generated beforehand in the sealedenvelop.com service using randomised permuted blocks of size 3, 6 and 9. The list was then transferred to REDCap.

Participants have equal likelihood of assignment to each condition (33%). They will not be blinded to allocation because the nature of the intervention does not allow it. They will be informed of their allocated treatment after completing the baseline questionnaires. Allocation will be concealed to the researchers at participant assignment and data analysis. The RR in-person workshop facilitator does not need to be blinded to allocation as the other two arms do not have a facilitator.

### Study participants

#### Inclusion criteria

Participant inclusion criteria include:

1. Being a mother with primary custody of a child/children aged 6–12 years
2. Residing in the Metro Vancouver Regional District,
3. Being able to speak, read and understand English

#### Participant recruitment

Participants will be recruited through advertising on online forums and social media, distributing notices through our networks, snowball sampling and posting notices in community centres.

Interested participants link to REDCap where they are provided with a comprehensive cover letter describing the study procedures and informing participants that completing the survey questions indicates consent. Once eligibility questions are answered, enrolled participants will be sent a link to the baseline questionnaire package to be completed in REDCap.

To promote participant retention and complete outcome data, a CDN$30 honorarium will be paid at baseline (T1) and CDN$15 at each follow-up (T2 and T3) as compensation for participation. Non-respondents will receive two email reminders to complete survey data. Participants attending in-person RR workshops are provided with an additional CDN$30 honorarium to compensate them for any expenses incurred in attending, such as travel or childcare.

#### Sample size considerations

The Tolerance for Risk in Play Scale (TRiPS) is our main study outcome. The TRiPS is scored on a logit scale and we know from previous data collection among parents of 5–13-year-old children that scores on the TRiPS range from 0.20 to about 1.95 with standard deviations in the range of 1.78 to 1.82 [45]. With a sample size of 81 mothers in each condition, a test that averaged the differences in TRiPS score from baseline to the first assessment will have 80% power at a 0.05 level of significance to detect a difference of 0.75 with the control condition when the standard deviation is 1.82 and the correlation between repeated observations is 0.75. From our previous work [46], we expect needing to complete baseline assessments among 501 mothers who will then be randomised into the three conditions. From our previous work [46], we are assuming a 65% retention rate at our first assessment (*n* = 325) and a 75% retention rate at our second assessment, which would result in a final sample of 244 mothers, corresponding to 81 in each condition.

### Intervention

#### RR digital tool

We adapted Bundy and colleagues’ RR in-person workshop [21, 36] using social cognitive theory [41] to incorporate health behaviour-change techniques (BCT) [40] that were amenable to a stand-alone online platform that was efficient, taking little time to understand and use, and would not require repeated visits to the tool [47]. For example, the use of associations, and reward and threat were not deemed possible as they would require an external assessor and/or repeat visits to the tool. We sought to address common concerns about risky play and engage participants in self-reflection tasks to consider how these concepts applied to their parenting approach. The participant proceeds through three chapters, as outlined in Table 1. The tool takes 15–45 min to complete, depending on participants’ movement through each task. Table 1 also outlines the BCT and social cognitive theory constructs that correspond to each task. Not reflected in the table are the following social cognitive theory constructs: social support, normative beliefs, and reinforcement and punishment. Social support (along with BCT 3.3 Social support (emotional)) is being addressed via encouraging participants to share the digital tool with their co-parent and social networks to promote discussion and change. Related to this, sharing the digital tool with their network may help prompt a shift in normative beliefs about risky play among their peer group. Reinforcement and punishments are not addressed because the tool involves interaction at one time point and these constructs would emerge after mothers try to make changes. For example, a punishment could be that their child is injured while engaged in risky play; a reinforcement could be that their child seems more confident since having more opportunities for risky play. Additional file 2 includes a description of measures we are using to assess social cognitive theory constructs. Interested readers are also directed to Michie et al.’s [40] BCT taxonomy for descriptions of each BCT, and to Glanz et al. [48] for a full description of social cognitive theory’s application to health-behaviour change.

**Table 1:** Risk-reframing (RR) intervention content, behaviour-change technique (v1) and social cognitive theory construct

RR intervention tasks	Behaviour-change technique^a^	Social cognitive theory construct
Home page Information and short video about risky play and why it is important, description of the tool components, logos of study partners	5.1 Information about health consequences 5.3 Information about social consequences 5.6 Information about emotional consequences 9.1 Credible source	• Outcome expectations • Knowledge
Chapter 1: Reflection 1. Selecting a child who will be the focus of the tasks 2. Values and traits most desired for the child in adulthood 3. Child’s favourite activities 4. Participant’s own favourite childhood activities 5. What the participant got out of these childhood activities 6. How do her child’s activities compare to what the participant remembers doing at that age?	13.2 Framing/reframing 13.3 Incompatible beliefs	• Outcome expectations • Knowledge
Chapter 2: What Would You Do? Participant is presented with three interactive video segments where she chooses to either allow or not allow the child to engage in the activity. Once the choice is made the rest of the video plays with the results of that choice. She can also see the results of the other choice, if she likes. The three scenarios involve: 1. Climbing a tree 2. Walking home from school 3. Building a fort	1.2 Problem solving 5.1 Information about health consequences 5.3 Information about social and environmental consequences 5.6 Information about emotional consequences 6.1 Demonstration of behaviour 9.3 Comparative imagining of future outcomes 13.2 Framing/reframing 15.3 Focus on past successes	• Outcome expectations • Knowledge • Observational learning • Barriers and opportunities • Self-efficacy
4. Common concerns: participant chooses from a list of fears that affect her in situations like the video scenarios (e.g. ‘ am concerned my child is going to get seriously hurt.’) 5. Things that helped me let go: participant chooses from a list of things that helped her let her child keep going in situations like the video scenarios (e.g. ‘It is important to me that my child has opportunities to learn, build skills and try new challenges.’)		
Chapter 3: Creating Your Plan 1. Participant revisits the values and traits she wanted most for her child when they grow up, and is prompted to think about what she is doing to promote those things, and whether there is anything she wants to change 2. Setting goals: participant is prompted to set one realistic and doable goal. Sample goals are provided 3. Steps I would take to achieve my goal: participant is prompted to consider graduated steps to achieve the goal. Sample steps are provided 4. I will begin my plan: participant sets a date for beginning her action plan 5. Participant is invited to print out or email herself a PDF version of her plan	1.1 Goal setting (behaviour) 1.2 Problem solving 1.3 Goal setting (outcome) 1.4 Action planning 6.1 Demonstration of behaviour 7.1 Prompts/cues 8.7 Graded tasks 9.3 Comparative imagining of future outcomes 13.2 Framing/reframing 13.3 Incompatible beliefs	• Outcome expectations • Knowledge • Observational learning • Barriers and opportunities • Self-efficacy • Behavioural skills • Intentions

^a^The behaviour-change technique (BCT) numbers in this column correspond with numbering in Michie et al.’s BCT taxonomy [40]

#### RR in-person workshop

The RR in-person workshop is a 2-h, facilitator-guided discussion of the same tasks as outlined in Table 1 above. Participants are taken through each task using PowerPoint slides that include the videos from the digital tool. The facilitator guide contains detailed guidance on discussion for each component and length of time to be dedicated to each slide. Participants are provided with a paper booklet to complete that mimics the online tasks.

For this RCT, a professional facilitator will be running all the workshops to ensure consistency in delivery. She does not have any prior knowledge or expertise on the topic, nor will she be involved in collecting or analysing the data. This was a deliberate choice in order to test real-world conditions where organisations with little or no background knowledge may be running the workshop, and ensure that our materials are sufficient to account for this. The workshops will include 6–12 participants and will be run once the minimum number of participants are enrolled for a given session.

#### Control condition

Participants in the control condition are presented with the Canadian Position Statement on Active Outdoor Play [43, 44]. The Position Statement is available at http://stage.participaction.com/sites/default/files/downloads/Participaction-PositionStatement-ActiveOutdoorPlay.pdf. This four-page document summarises the issues and research regarding children’s access to outdoor play and provides recommendations for various stakeholders. It was developed by a cross-sectoral consortium of researchers, practitioners and stakeholders. It states that ‘Access to active play in nature and outdoors – with its risks – is essential for healthy child development’ and recommends increasing children’s opportunities for self-directed play in all settings [43, 44]. The Position Statement includes recommendations for parents, educators, health professionals, administrators and various levels of governments to address the barriers to children’s outdoor play. Recognising the role of widespread risk aversion in limiting play, the Position Statement addresses common misconceptions and encourages that danger be differentiated from risk and outdoor play and fun be valued as much as safety.

### Study data

#### Measurement occasions and follow-up

Participants will complete a questionnaire package at three time points: baseline (T1), 1 week post intervention (T2) and 3 months post intervention (T3). Long-term change is unlikely if participants do not make initial changes, thus, the 1-week follow-up was selected to assess short-term effectiveness, while still providing participants sufficient time to make their initial planned changes. The 3-month follow-up will assess long-term effectiveness once participants have had several months to reflect on the intervention and implement change.

**Fig. 2:**
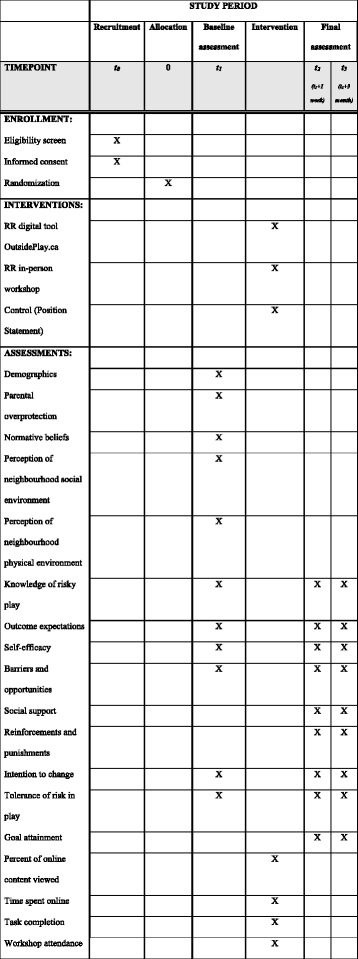
Schedule of enrollment, intervention and assessments according to Standard Protocol Items: Recommendations for Interventional Trials (SPIRIT)

Survey data will be collected and managed using REDCap. See Fig. 2, the Standard Protocol Items: Recommendations for Intervention Trials (SPIRIT) schedule, for an overview of the study schedule and measures.

Baseline data collection includes socio-demographic data: age, ethnicity, marital status, education, employment, home dwelling type, household income and number of children in the household. Participants will also complete measures to assess primary and secondary outcomes at each time point.

#### Outcome measures

The primary outcome measure is increase in the total score on the Tolerance of Risk in Play Scale (TRiPS), a 31-item measure examining adults’ tolerance of risk during children’s play, which has been psychometrically validated [45]. The scale is based on Sandseter’s six-category model of risky play [9]. Goodness-of-fit statistics for TRiPS have been found to be in the acceptable range [45]. Examination of logical item hierarchy indicated that items that were relatively difficult to endorse (e.g. ‘Would you let the child play near the edge of steep cliffs?’) were located higher on the hierarchy than those that were easier to endorse (e.g. ‘Would you let the child play in the backyard supervised?’). The Person Separation Index was 2.63, indicating that the measure separated persons into more than two distinct groups, such as more and less risk tolerant. The Person Reliability Index was 0.87 indicating that the instrument was able to consistently differentiate between those scoring high versus low. Self-perceived risk tolerance was highly positively associated with scores on TRiPS, and the mean score increased with age of the child [45].

The secondary outcome measure is self-reported behaviour change*.* Behaviour change is being measured by their self-reported progress on attaining the goal they set for themselves within the RR tool. Participants will be reminded of their goal and asked: ‘Did you accomplish your goal?’ with ‘Yes’ and ‘No’ response options.

#### Adherence to intervention

Adherence to the RR digital tool will be measured by examining the percentage of content viewed, time spent online and task completion [49]. Adherence to the in-person RR tool will be measured by examining workshop attendance and task completion. Further outcomes and measures are described in detail in Additional file 2. All measures that were created for this study can be seen in Additional file 3.

### Data management

Data will be entered by participants directly into REDCap, which is hosted on a secure, firewall-protected server at British Columbia Children’s Hospital Research Institute. The database is password protected and only accessible by responsible staff. REDCap maintains an audit trail that captures all user activity, including data manipulation and export. Exported data will be stored on a secure, firewall-protected server at British Columbia Children’s Hospital Research Institute in a password-protected file only accessible by responsible staff.

### Statistical analysis

Analysis strategy (including verification of model assumptions) will follow Singer and Willet’s guidelines [50]. All participants allocated to one of the three conditions will be included in the analysis, regardless of deviation from protocol, missed follow-up observations, or withdrawal. To test our hypotheses that mothers completing either version of the RR tool will have significantly greater increase of tolerance for risk in play, we will compare the two intervention conditions with the control condition. For modelling purposes, we will use mixed-effects models using a correlation structure that assumes model change over time. Selection of the most appropriate model will depend on the distributional form of the data, whether the change is linear and non-linear and model selection will be based on residual analyses.

To test our hypotheses that a higher proportion of mothers in either version of the RR tool will report attainment of behaviour-change goals (secondary outcome measure), we will use logistic regressions.

#### Missing data

We will use multiple imputation to manage missing data and will report and justify our imputation strategies. Imputed data for multiple imputation will be analysed as part of a sensitivity analysis.

#### Statistical software

The latest version of R (R Foundation for Statistical Computing, Vienna, Austria) and Stata (StataCorp LLC, College Station, TX, USA) will be used for statistical analysis and graphics.

### Quality assurance and monitoring

Written standard operating procedures are used for all study procedures to ensure data quality and consistent application of study protocols. State of recruitment, data completeness, control of correct randomisation and allocation of participants is regularly verified. Any deviations from expected standards will be reported to, and discussed with, the project manager. Any protocol modifications will be reported to the University of British Columbia/Children’s and Women’s Health Centre of British Columbia Research Ethics Board and the trial registry, the United States National Institute of Health’s Protocol Registration and Results System.

### Ethical considerations

The health risks of the RR interventions are negligible. The potential benefits are that participants learn more about the importance of children’s outdoor risky play. Also, potential benefits could come from changes in parenting practices that allow the child to engage in more risky play. The study has been approved by the University of British Columbia/Children’s and Women’s Health Centre of British Columbia Research Ethics Board (Certificate #H15–03271).

## Discussion

Children’s engagement in risky play has been associated with a multitude of developmental, physical and mental health outcomes. Despite this, children’s opportunities for risky play show steady decline across generations [17–19]. Parent fears and exaggerated perceptions of risks, such as abduction and traffic injury, are a major deterrent to children’s engagement in risky play [20–28]. The RR tool seeks to address and reframe parents’ beliefs about risk and shift their parenting approach. Using established behaviour-change techniques grounded in social cognitive theory, the RR tool provides an innovative evidence-based, rigorously designed method to influence individual and societal views on children’s risky play. The digital tool can be easily and widely shared for broad reach, and the in-person workshop can be integrated into the pre-existing curriculum of organisations that work with parents.

This study will comprehensively evaluate the effectiveness of the digital and in-person workshop versions of the RR tool. It will also add to the understanding of the potential effectiveness of digital technology in influencing parental attitudes and behaviours with regard to risky play. The findings will provide rich data to inform widespread RR efforts to increase opportunities for outdoor risky play among children.

### Trial status

Recruitment for the study began on 30 November 2017 and is anticipated to be completed by 31 December 2018.

## Additional files

Additional file 1: Standard Protocol Items: Recommendations for Interventional Trials (SPIRIT) Checklist. (DOC 121 kb)

Additional file 2: Description of study measures and descriptions. (DOCX 114 kb)

Additional file 3: Measures created for this study. (DOCX 101 kb)

## Abbreviations

BCT: Behaviour-change technique
RCT: Randomised controlled trial
RR: Risk-reframing
SPIRIT: Standard Protocol Items: Recommendations for Intervention Trials
TRiPS: Tolerance of Risk in Play Scale
