# Supplementary material for: Go Play Outside! Effects of a risk-reframing tool on mothers’ tolerance for, and parenting practices associated with, children’s risky play: study protocol for a randomized controlled trial
Source: Trials. 2018 Mar 7;19:173. doi: 10.1186/s13063-018-2552-4 (PMC5842626; doi:10.1186/s13063-018-2552-4)
Supplement: Supplementary file 2 — Description of study measures and descriptions. (DOCX 114 kb) [file 13063_2018_2552_MOESM2_ESM.docx]

**Electronic supplementary material**

**Study data – further outcomes**

The following measures are collected at baseline, 1 week and 3 months post intervention.

**Social Cognitive Theory Constructs**

**Knowledge**. We developed a measure of perceptions of risky play to assess the participant’s knowledge and belief that allowing children to engage in risky play will have positive outcomes. It includes 10 items measured on a 5-point scale from “strongly disagree” to “strongly agree.” The participant is asked to rate the extent to which they agree that “Risky play can help a child:”, with items including: “learn to make decisions”, and “develop skills to manage risks”.

**Outcome expectations**. The Positive Potentiality of Risky Play measure described above was altered such that the participant was asked to rate the extent to which they agree that “Risky play can help my child,” as opposed to “a child.” All other aspects are the same.

**Self-efficacy and behavioural skills**. Participants complete 4 items on a 5-point scale from “strongly disagree” to “strongly agree” assessing their belief in their ability to provide their child with opportunities for risky play, resisting the urge to intervene when their child is engaged in risky play, convincing their co-parent to support their intentions, and being able to maintain long-term change.

**Social support**. Participants are asked whether they have shared the online RR tool with their co-parent (yes/no), and to rate on a 5-point scale the extent to which their co-parent sees the benefits of risky play, they have talked to their co-parent about risky play and made plans for change together.

**Barriers and opportunities**. Participants use a four-point scale ranging from “never” to “often” to rate the extent to which four concerns (injury, harm by others, social censure, lack of confidence in child’s abilities to stay safe) make it difficult for them to let their child engage in risky play. The same scale is used to rate the extent to which four beliefs (trust in child, want to support child’s desire, want to give child opportunity to build skills, see learning opportunity) help them let their child engage in risky play.

**Normative beliefs.** We adapted a scale used in previous research [1] to measure normative beliefs regarding how normal it is for children in the participant’s neighbourhood to engage in ten risky play behaviours, as well as how normal it is for parents to be criticized for letting their child do risky play and for children to get in trouble. It is rated on a 5-point scale ranging from “strongly disagree” to “strongly agree.”

**Reinforcements and punishments.** At follow-ups, we will be measuring five positive and five negative events that participants experienced in the interim resulting from their child’s engagement in risky play. Sample positive events are that their child seems more confident or more physically active; sample negative events are their child experiencing an injury or being criticized by others for letting their child engage in risky play. These are rated on a four-point scale ranging from “strongly disagree” to “strongly agree”.

**Additional data**

**Overprotection**. The Parental Overprotection Measure [2,3] will be completed at baseline to assess mothers’ tendency for overprotection. Items are completed on a 5-point scale ranging from “not at all” to “very much” and ask parents to select the number that represents their usual response to their child. Sample items include “I anticipate and avoid situations where my child might do something risky” and “I shelter my child from life’s difficulties.”

**Parent anxiety**. The Depression Anxiety Stress Scales 21 (DASS-21) [4,5] is included to measure baseline anxiety. It includes three seven-item scales that measure self-reported depression, anxiety and stress over the past week on a four-point scale, ranging from “0 did not apply to me at all” to “3 applied to me very much or most of the time.” Cronbach’s alpha was 0.93 in a non-clinical community sample [4]. The DASS-21 also shows good convergent and discriminant validity when compared with other measures of depression and anxiety [4].

**Perception of neighbourhood social and physical environment.** The Neighbourhood Relations Scale [6] includes five items rated on a five-point scale from “every day” to “never” that measures frequency and types of interaction with neighbours. We are also including two scales from the Neighbourhood Environment Walkability Scale [7]: Neighbourhood safety and Crime safety. Measured on a five-point scale ranging from “strongly disagree” to “strongly agree”, items assess elements such as perceptions of density of traffic and pedestrian safety, and their worries about letting their child out.

**Intention to change.** Immediately after completing the intervention, participants complete two questions. On a four-point scale ranging from “strongly disagree” to “strongly agree,” participants rate whether they think the benefits of risky play outweigh the harms. They also select one statement from five statements assessing their intention to change (e.g., “I have no intention of letting my child do risky play”).

**References**

1. Duncan S, McPhee J. State of play survey: Executive report. Auckland, NZ; 2015.

2. Clarke K, Cooper P, Creswell C. The Parental Overprotection Scale: Associations with child and parental anxiety. J. Affect. Disord. 2013;151:618–24.

3. Edwards SL, Rapee RM, Kennedy S. Prediction of anxiety symptoms in preschool-aged children: Examination of maternal and paternal perspectives. J. Child Psychol. 2010;51:313–21.

4. Henry JD, Crawford JR. The short-form version of the Depression Anxiety Stress Scales (DASS-21): Construct validity and normative data in a large non-clinical sample. Br. J. Clin. Psychol. 2005;44:227–39.

5. Antony MM, Bieling PJ, Cox BJ, Enns MW, Swinson RP. Psychometric properties of the 42-item and 21-item versions of the Depression Anxiety Stress Scales in clinical groups and a community sample. Psychol. Assess. 1998;10:176–81.

6. Prezza M, Amici M, Roberti T, Tedeschi G. Sense of community referred to the whole town: Its relations with neighboring, loneliness, life satisfaction, and area of residence. J. Community Psychol. 2001;29:29–52.

7. Rosenberg D, Ding D, Sallis JF, Kerr J, Norman GJ, Durant N, et al. Neighborhood environment walkability scale for youth (NEWS-Y): Reliability and relationship with physical activity. Prev. Med. 2009;49:213–8.

8. Donkin L, Christensen H, Naismith SL, Neal B, Hickie IB, Glozier N. A systematic review of the impact of adherence on the effectiveness of e-therapies. J. Med. Internet Res. 2011;13:e52.
