# Supplementary material for: Go Play Outside! Effects of a risk-reframing tool on mothers’ tolerance for, and parenting practices associated with, children’s risky play: study protocol for a randomized controlled trial
Source: Trials. 2018 Mar 7;19:173. doi: 10.1186/s13063-018-2552-4 (PMC5842626; doi:10.1186/s13063-018-2552-4)
Supplement: Supplementary file 3 — Measures created for this study. (DOCX 101 kb) [file 13063_2018_2552_MOESM3_ESM.docx]

**Secondary outcome measure: Goal attainment**

At the beginning of the study, you set a goal for yourself regarding something you wanted to change to give your child more opportunities for risky play. Did you accomplish your goal? (Scale: Yes/No)

**Knowledge of risky play**

Scale: 1=strongly disagree, 5=strongly agree

*Risky play is thrilling and exciting play that gives children opportunities for challenging themselves, testing their limits, and exploring boundaries; for example, climbing as high as s/he likes, play-fighting, building forts with tools, exploring the neighbourhood without an adult. Please select the answer that best applies to you.*

*Risky play can help a child…*

1. Learn to make decisions.
2. Develop skills to manage risks.
3. Develop social skills.
4. Build resilience.
5. Develop self-esteem.
6. Develop self-confidence.
7. Be more physically active.
8. Build courage.
9. Become more curious about the world.
10. Become more imaginative.

**Outcome expectations**

Scale: 1=strongly disagree, 5=strongly agree

*Now think about your child specifically to answer the following questions.*

*Risky play can help* ***my child****:*

1. Learn to make decisions.
2. Develop skills to manage risks.
3. Develop social skills.
4. Build resilience.
5. Develop self-esteem.
6. Develop self-confidence.
7. Be more physically active.
8. Build courage.
9. Become more curious about the world.
10. Become more imaginative.

**Self-efficacy and behavioural skills**

Scale: 1=strongly disagree, 5=strongly agree

*When it comes to letting my child do risky play…*

1. I could find ways to give my child more opportunities for risky play.
2. I could resist the urge to intervene when my child does risky play.
3. I could convince my partner/co-parent to support my intention to let my child do more risky play.
4. I could maintain change in the long-term to let my child do risky play.

**Social support**

1. I have shared OutsidePlay.ca with my partner/co-parent. (Scale YES/NO)

Scale: 1=Not at all, 5=A great deal

1. My partner sees the benefits of risky play.
2. We have talked about how to let our child do more risky play.
3. We have made plans to let our child do more risky play.

**Barriers and opportunities**

Scale: 1=Never, 4=Often

*To what extent do these concerns make it difficult for you to let your child do risky play?*

1. I am concerned that my child is going to get seriously hurt.
2. I think my child does not know how to stay safe.
3. I am worried that someone is going to harm my child, either kidnapping or assault by an adult, or bullying by other children.
4. I am concerned that other people are going to think I am a bad parent for letting my child take risks.

*To what extent do these beliefs help you let your child do risky play?*

1. I have seen my child manage risky play like this before and trust s/he can handle it.
2. I believe my child wants to do risky play and I want to give him/her my support.
3. It is important to me that my child has opportunities to learn, build skills and try new challenges.
4. I think risky play is a good learning opportunity for my child.

**Normative beliefs**

Scale: 1=strongly disagree, 5=strongly agree

*In my neighbourhood, it is normal for children to…*

1. Climb trees.
2. Jump from heights.
3. Play on playground equipment.
4. Engage in rough-and-tumble games (e.g., wrestling, play fighting).
5. Use adult tools (e.g., hammer, saw).
6. Use loose parts (e.g., sticks, tires, timber, tarpaulins) during outdoor play.
7. Roam the neighbourhood with friends but without an adult.
8. Roam the neighbourhood alone.
9. Ride bicycle or skateboard in the neighbourhood with friends but without an adult.
10. Ride bicycle or skateboard in the neighbourhood alone.

*In my neighbourhood, it is normal for….:*

1. Parents to be criticized for letting their child do risky play.
2. Children to get in trouble when they do risky play.

Scale: Likert 5 point (1=strongly disagree, 5=strongly agree; N/A=I did not provide my child with more opportunities for risky play)

**Reinforcements and punishments**

Scale: 1=strongly disagree, 5=strongly agree; N/A=I did not provide my child with more opportunities for risky play

**Reinforcements**

1. My child is happier when I let him/her do risky play.
2. My child is more physically active since having more opportunities for risky play.
3. My child seems more confident since having more opportunities for risky play.
4. My child seems more able to handle things by him/herself since having more opportunities for risky play.
5. My child seems less afraid since having more opportunities for risky play.

**Punishments**

1. My child was hurt while doing risky play.
2. My child had a bad experience while doing risky play.
3. I was criticized by friends or neighbours for letting my child do risky play.
4. My child got in trouble at school or a recreation facility for doing risky play.
5. I got in trouble with school staff for allowing my child to do risky play.

**Intention to change**

Scale: 1=strongly disagree, 5=strongly agree

- I think the benefits of risky play outweigh the potential harms.

*Which of these statements best reflects your situation?*

- I have no intention of letting my child do risky play.
- I intend to make some changes in the next week to let my child do risky play.
- In the past week, I have already made some changes to let my child do risky play.
- I don’t need to make any changes because my child already does enough risky play.
- I made changes to allow my child to do risky play, but now I am back to the way things were before.
